# Effects of Short-Term Exposure to High-Dose Inhaled Corticosteroids on Appetite, Dietary Intake, Leptin Levels, and Body Weight in Adults with Asthma—A Prospective Pilot Study

**DOI:** 10.3390/jpm15070326

**Published:** 2025-07-20

**Authors:** Sotirios Kakavas, Dimitrios Karayiannis

**Affiliations:** 1 Intensive Care Unit, Henry Dunant Hospital Center, 11526 Athens, Greece; 2 Department of Clinical Nutrition, Evaggelismos General Hospital, 10676 Athens, Greece; dkarag@hua.gr

**Keywords:** inhaled corticosteroids (ICSs), asthma exacerbations, appetite changes, dietary intake, body weight

## Abstract

**Background:** Inhaled corticosteroids (ICSs) are a cornerstone in asthma management, particularly during exacerbations, when high doses are often prescribed. However, patient concerns about potential side effects such as increased appetite, weight gain, and metabolic disturbances may reduce adherence, compromising treatment outcomes. While oral corticosteroids (OCSs) are well known to induce such effects, the metabolic impact of short-term high-dose ICSs remains poorly studied. **Objective:** This prospective pilot study aimed to assess whether a 14-day course of high-dose ICSs in adults with stable asthma induces changes in appetite, dietary intake, leptin levels, or body weight. **Methods:** Thirty-five adults (19 males, 16 females; mean age 48.7 ± 15.1 years) with stable mild asthma received ≥400 µg/day extrafine beclomethasone dipropionate/formoterol via pressurized metered-dose inhaler for 14 days. Participants underwent assessments at baseline and after 14 days, including body weight, BMI, fasting serum leptin levels, dietary intake (evaluated using 24 h dietary recalls), and appetite (measured via a visual analogue scale). **Results:** No significant changes were observed in body weight (mean change: −0.38 kg; 95% CI: −0.81 to 0.05; *p* = 0.083) or BMI (*p* = 0.912) following high-dose ICS use. Similarly, serum leptin levels (mean change: 0.13 ng/mL; 95% CI: −3.47 to 3.72; *p* = 0.945), subjective appetite scores (mean change: −4.93 mm; 95% CI: −13.64 to 3.79; *p* = 0.267), and dietary energy intake (mean change: +255 kJ/day; 95% CI: −380 to 891; *p* = 0.431) did not differ significantly post-intervention. **Conclusions:** Short-term high-dose ICS therapy in adults with mild asthma may not significantly affect appetite, dietary intake, leptin levels, or body weight. These findings support the metabolic safety of short-term high-dose ICSs and may help alleviate patient concerns, improving adherence during exacerbation management.

## 1. Introduction

Asthma is a prevalent chronic inflammatory airway disease, with inhaled corticosteroids (ICSs) being the most widely prescribed controller medications [1]. Despite their effectiveness in managing symptoms and reducing exacerbations, the use of ICSs is often limited by their side effect profile, which includes suppression of the hypothalamic–pituitary–adrenal (HPA) axis, reduced growth velocity in children, osteoporosis, and increased susceptibility to respiratory infections [1,2]. These systemic effects arise from both the fraction of the drug that is swallowed and absorbed through the gastrointestinal tract (which bypasses first-pass hepatic metabolism) and the fraction absorbed directly through the pulmonary vasculature.

Although ICSs are generally considered to have low systemic bioavailability, adverse effects are reported in a significant proportion of users [1]. It is important to note that higher drug potency does not always translate into greater clinical efficacy. Even small amounts of ICSs may lead to systemic absorption and undesirable effects [1]. The risk is especially elevated in children and adults with severe asthma and increases with dose. Although not definitively proven, high-dose ICSs are also suspected of causing metabolic disturbances, including elevated blood glucose levels, increased appetite, and weight gain [3]. These effects, although often viewed as minor from a medical standpoint, can significantly affect patient-reported outcomes, adherence to therapy, and overall quality of life [4,5].

To date, the literature primarily focuses on severe adverse events associated with prolonged high-dose ICS use, while reports on metabolic effects are largely anecdotal [1,2,3]. Nevertheless, in clinical practice, short courses of high-dose ICSs are frequently used to manage moderate-to-severe asthma exacerbations [2]. While oral corticosteroids (OCSs) are known for their more pronounced systemic effects, patients also express concerns about weight gain, increased appetite, and dysregulated glucose metabolism with high-dose ICS use. These concerns may impact adherence, particularly when patients perceive a mismatch between symptom relief and undesirable side effects [4,5]. Currently, there is a paucity of evidence directly examining the relationship between ICSs and metabolic effects such as weight gain and increased appetite. A systematic review investigating short-term OCS use found limited evidence supporting their role in causing weight gain, increased appetite, or elevated energy intake, and called for further investigation [6].

Asthma is a heterogeneous inflammatory disease involving multiple immune pathways, with eosinophils, T-helper 2 (Th2) cells, and cytokines such as interleukin (IL)-4, IL-5, and IL-13 serving as central mediators of allergic and eosinophilic asthma phenotypes [6]. Exacerbations—acute episodes typically triggered by environmental factors such as respiratory viruses, allergens, or pollutants—vary in severity, with some resolving spontaneously and others requiring emergency interventions or hospitalization [2]. ICSs play a central role in minimizing the risk and severity of exacerbations, and treatment guidelines often recommend a short-term increase in ICS dose at the first signs of worsening symptoms, particularly when guided by written asthma action plans [2]. This approach is used to avert the need for systemic corticosteroids and reduce the likelihood of hospital admission.

Additionally, there is a well-documented association between increased body mass index (BMI), obesity, and asthma. Obese individuals are not only at higher risk of developing asthma but also tend to experience more severe disease, reduced responsiveness to standard therapies, and a lower quality of life [7]. The pathophysiological mechanisms underpinning this link remain incompletely understood but may involve adipokines such as leptin and other inflammatory cytokines secreted by adipose tissue [8,9]. Whether these mediators are pathogenic or serve as useful biomarkers for disease activity and treatment response remains unclear.

Interestingly, OCSs have been shown to increase appetite, body weight, and circulating levels of leptin and adiponectin in patients with asthma and chronic obstructive pulmonary disease (COPD) [10,11]. However, the metabolic effects of short-term high-dose ICSs—particularly their impact on appetite regulation, weight, and systemic adipokine levels—have not been systematically studied.

## 2. Objective

Given the lack of robust evidence, it is essential to determine the likelihood and extent of metabolic side effects associated with short-term high-dose ICS therapy in asthma. Gaining insight into these effects may help improve treatment adherence and inform targeted strategies to manage potential increases in appetite and weight. Therefore, the aim of this study was to assess whether short-term high-dose ICS treatment in adults with stable asthma is associated with significant metabolic changes, including alterations in leptin and glucose levels, dietary intake, and appetite.

## 3. Methods

### 3.1. Study Design

**Figure 1:**
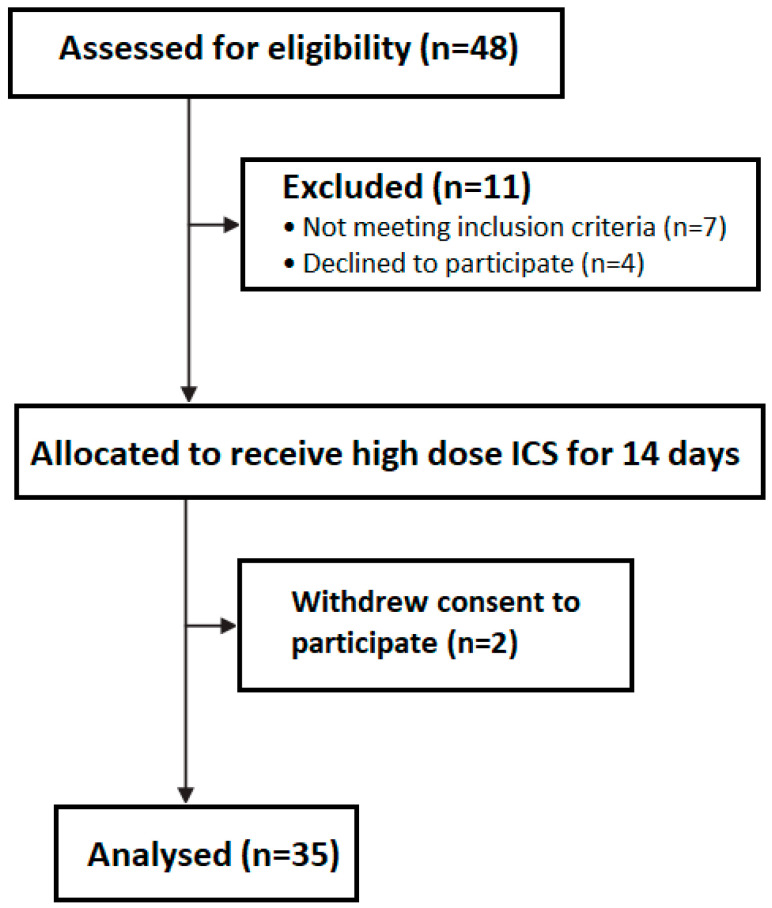
Consort participant flow diagram.

The subjects of the present study were 35 outpatients (16 men, 19 women) with a previous diagnosis of adults with mild asthma, as defined by previous treatment allocation to GINA steps 1 or 2, who were recruited from general practices and a hospital outpatient clinic (Figure 1). As this was an exploratory pilot study, the sample size was determined pragmatically based on feasibility and literature suggesting that a minimum of 30 participants is generally sufficient to estimate variability in key outcomes for future sample size planning. Inclusion criteria included age ≥18 years, confirmed diagnosis of asthma, and no recent use of oral corticosteroids (OCSs) within the past 6 weeks. Asthma diagnosis was confirmed based on medical history of episodic respiratory symptoms, spirometric reversibility (≥12% and ≥200 mL improvement in FEV1 post-bronchodilator), or historical bronchial hyperresponsiveness to methacholine. All patients were previously treated according to GINA [2] steps 1 or 2, receiving either as-needed low-dose ICS–formoterol or as-needed short-acting beta-agonist (SABA) combined with low-dose ICSs. Patients at GINA step 2 also received low-dose ICSs as regular maintenance therapy. Participants were steroid-naïve with respect to medium or high-dose ICSs, and were excluded if they required OCSs within 6 weeks prior to the study or had already escalated ICS doses during recent self-management of asthma worsening. All patients were mildly symptomatic at screening, reporting one or more of the following: wheezing, chest tightness, or exertional dyspnea, with peak expiratory flow (PEF) >60% of predicted or personal best consistent with non-severe asthma worsening. The present study was approved by the Research Ethics Committee of the “Evangelismos” General (Approval Code: 465 Approval Date: 11 January 2023). At the time of recruitment, an informed written consent was obtained by all study participants. This study is fully compliant with the tenets of the WMA Declaration of Helsinki—Ethical Principles for Medical Research Involving Human Subjects.

### 3.2. Study Subjects

After confirming eligibility, all participants were placed on maintenance therapy with extrafine beclomethasone dipropionate/formoterol (BDP/F 100/6 µg), two inhalations twice daily. They were instructed to increase dosing as needed (up to 8 inhalations/day), allowing them to reach a high daily dose of ≥400 µg of BDP during the 14-day intervention period.

Patients were informed to seek emergency care in the event of worsening symptoms, inability to perform daily activities, or if peak flow did not improve after 24 h despite rescue inhaler use. Subjects were reassessed at day 14 (visit 2).

### 3.3. Clinical Intervention

Eligibility screening was performed prior to inclusion. At visit 1, eligible subjects were examined by a pulmonologist and a dietary assessment was performed. Patients were placed on maintenance therapy with two inhalations of extrafine BDP/F (100/6 μg) twice daily, and they were also instructed to increase BDP/F as needed (maximum daily dose is 8 inhalations). In this way, subjects reached high doses of ICSs (>400 μg for beclometasone) for the next 2 weeks [2]. The subjects were informed that they should seek emergency care in case of any of the following: intense or worsening dyspnea;inabilityto talk, walk, or complete usual daily activities; persistent or worsening symptoms or deteriorating PEF after 24 h; unimproved symptoms despite the use of rescue medications;or blue lips or fingernails. Subjects were reassessed after 14 days at visit 2. Patients included in the study were mildly symptomatic at baseline and presented with features consistent with early or moderate asthma worsening (e.g., increased use of relievers, daytime symptoms, nocturnal awakening). According to GINA guidelines, early increases in ICS dose are recommended as part of individualized asthma action plans to prevent exacerbations and reduce the need for systemic corticosteroids. Therefore, a temporary step-up to high-dose ICSs was clinically justified in all cases.

### 3.4. Measures

At baseline (visit 1), basic demographic data were obtained with a structured clinical history and physical examination. Recorded data included age, sex, lung function test results, number of acute exacerbations in the past year, and number of hospitalized exacerbations in the past year. In order to measure the adequacy of asthma control, a validated reduced version of the Asthma Control Questionnaire (ACQ) without spirometry or peak expiratory flow rate testing was used. This version consists of six items measured on a 7-point scale, from 0 to 6, during a recall period of the past 7 days [12]. The computed mean score of the six items ranges from 0 (no impairment) to 6 (extreme impairment). Participants were especially queried on the previous use of ICSs, OCSs, and/or antibiotics. Whenever possible, patient reports were verified with medical records. All subjects were assigned to receive maintenance therapy with two inhalations of extrafine BDP/F (100/6 μg) twice daily, and they were also instructed to increase BDP/F as needed (maximum daily dose is 8 inhalations). A second visit was scheduled for after 2 weeks.

Body weight was assessed with electronic scales at each study visit, with a precision of ±0.1 kg (SECA 769 scale). Standing height was measured using a wall-mounted stadiometer, with a precision of ±0.001 m. Body mass index (BMI) was computed as weight divided by the square of height (kg/m^2^). According to the World Health Organization (WHO) criteria, individuals with a BMI less than 18.5 kg/m^2^ were categorized as underweight, those with a BMI between 25.0 and 29.9 kg/m^2^ as overweight, and those with a BMI equal to or greater than 30.0 kg/m^2^ as obese. A 20 mL blood sample was collected on visits 1 and 2 from patients after at least a 12 h fast. Complete blood counts and biochemical values were measured. Plasma leptin was measured with an in-house RIA using a commercial kit (Linco Research, St. Louis, MO, USA). To assess the regular consumption of food and alcohol, each participant completed two 24 h dietary recalls to evaluate participants’ energy, macronutrient, and micronutrient intake. These recalls took place through face-to-face interviews during the patients’ assessments. The three-pass approach was employed in obtaining dietary recalls. To enhance the accuracy of portion size reporting, participants were instructed to indicate quantities of individual foods and beverages using common household objects (such as teaspoons, tablespoons, cups, etc.) and familiar items (such as matchboxes, cell phones, palms, etc.). In instances of complex food recipes or when standard measures were not applicable, a booklet featuring multiple photos of various portion sizes for various food items was utilized. Participants were then asked to specify their portion size based on the provided photos. Each dietary recall was analyzed using Nutritionist ProTM (Axxya Systems, Stafford, TX, USA). Subjective appetite sensation in fasting was assessed using a 10 cm visual analogue scale (VAS) [13]. Data were collected by a trained dietician.

### 3.5. Statistical Analysis

The assumption of normal distribution of continuous variables was assessed by the Kolgomorov–Smirnov criterion. In case of a normal distribution, quantitative variables were expressed as the means ± standard deviation. Otherwise, they were presented as medians and ranges (interquartile range), while categorical variables were expressed as percentages. Qualitative (categorical) variables were reported as absolute and relative frequencies. Comparisons of categorical variables were performed with the use of Pearson’s chi squared and Fisher’s exact tests. Student’s *t*-tests and Mann–Whitney tests were performed for the comparison of continuous variables between two groups. For comparisons of continuous normally distributed paired variables, the paired *t*-test was used. Wilcoxon signed ranks test was used for paired nonparametric variables. All reported *p* values are two-tailed, and statistical significance was set at *p* < 0.05. The elaboration and analysis of data were conducted using SPSS statistical software (version 26.0).

## 4. Results

A total of 35 adults with stable, mild asthma (19 males, 16 females; mean age 48.7 ± 15.1 years) completed the 14-day high-dose inhaled corticosteroid (ICS) intervention. Baseline demographic and clinical characteristics, including lung function (FEV_1_: 89 ± 5.9% predicted), asthma control (ACQ6: 1.7 ± 0.5), and body mass index, were comparable between genders (all *p* > 0.05) (Table 1). The mean BMI was 28.8 kg/m^2^, indicating that participants were, on average, overweight according to WHO criteria. Adherence to the prescribed ICS regimen was objectively verified through a statistically significant reduction in peripheral blood eosinophil counts following the intervention (mean decrease: −0.29 × 10^9^/L, 95% CI: −0.39 to −0.19, *p* < 0.001). Despite this, there were no statistically significant changes observed in fasting serum leptin concentrations (mean change: 0.13 ng/mL, 95% CI: −3.47 to 3.72, *p* = 0.945) or in subjective appetite scores as assessed by the visual analogue scale (VAS) (mean change: −4.93 mm, 95% CI: −13.64 to 3.79, *p* = 0.267) after the high-dose ICS period.

Furthermore, comprehensive analysis of dietary intake and anthropometric measures revealed no significant alterations attributable to the intervention. Total daily energy intake, as assessed by validated four-day food records and food frequency questionnaires, did not differ significantly between pre- and post-ICS treatment (mean difference: 255 kJ/day, 95% CI: −380 to 891, *p* = 0.431). Body weight remained stable throughout the study duration (mean change: −0.38 kg, 95% CI: −0.81 to 0.05, *p* = 0.083), with no significant shifts observed in BMI categories or in body composition parameters (Table 2). Subgroup analyses by sex and baseline BMI failed to identify any differential effects of ICSs on metabolic or appetite-related outcomes. Collectively, these findings suggest that a short-term course of high-dose ICSs in adults with mild asthma does not induce clinically or statistically significant changes in appetite, dietary intake, serum leptin levels, or body weight, supporting the metabolic safety profile of short-term high-dose ICS therapy in this patient population.

## 5. Discussion

This prospective pilot study offers valuable new insights into the metabolic effects of short-term high-dose inhaled corticosteroid (ICS) therapy in adults with stable asthma. We found that a 14-day course of high-dose extrafine beclomethasone dipropionate did not lead to significant changes in serum leptin concentrations, subjective appetite, dietary intake, or body weight. These findings are particularly relevant for asthma management, where concerns about potential weight gain and metabolic side effects often influence patients’ willingness to adhere to ICS regimens, especially during periods of dose escalation for exacerbation control.

Our findings align with the limited available evidence suggesting that ICSs, even at high doses, are unlikely to cause significant short-term metabolic effects [6,14]. In contrast, OCSs are welldocumented to increase appetite, promote weight gain, and elevate adipokine levels, including leptin and adiponectin, in patients with asthma [14]. However, robust data specifically examining the relationship between ICSs and metabolic outcomes—such as weight gain or appetite changes—remain scarce. A recent systematic review of short-term OCS use found limited evidence supporting their role in causing weight gain, increased appetite, or higher energy intake, highlighting the need for further research [6]. Our study helps address this gap by providing objective evidence regarding the metabolic effects of short-term high-dose ICSs on appetite, dietary intake, leptin levels, and body weight.

The absence of significant changes in leptin concentrations or subjective appetite ratings in our study suggests that short-term high-dose ICSs do not disrupt appetite regulation pathways mediated by adipose tissue. Leptin, an adipose-derived hormone, plays a central role in regulating appetite and energy balance [15]. While OCSs have been shown to acutely increase leptin levels—likely through direct effects on adipocytes and increased adiposity—our findings suggest that ICSs do not exert similar effects inthe short term. This distinction is clinically meaningful, as it indicates that the metabolic risks associated with short-term high-dose ICSs are minimal, particularly compared with OCSs.

Beyond leptin, our study evaluated dietary intake and body weight using validated food-frequency questionnaires and precise anthropometric measures. We found no significant changes in energy intake or body weight following the 14-day high-dose ICS intervention. These results are consistent with prior reports, which generally have not demonstrated significant short-term metabolic effects with ICS use. While some large-scale observational studies have linked long-term ICS use to modest weight gain (1.1–2.3 kg/year), these findings are often confounded by indication, recall bias, and the absence of objective dietary assessments [16]. Our study’s use of validated dietary tools and objective adherence verification via eosinophil suppression strengthens the credibility of our findings.

The clinical relevance of these results is reinforced by current asthma guidelines, which recommend short-term increases in ICS dose at the onset of exacerbations to reduce reliance on systemic corticosteroids and lower hospitalization risk. The reassurance that short-term high-dose ICSsare unlikely to significantly impact appetite or body weight may help improve patient adherence to guideline-recommended therapies. This is especially important because concerns about weight gain and metabolic disturbances are frequently cited as barriers to ICS adherence.

It is also important to consider these findings in the broader context of asthma, obesity, and metabolic health. A well-established association exists between increased body mass index (BMI), obesity, and greater asthma severity [17,18,19,20]. Individuals with obesity not only face a higher risk of developing asthma but also often experience more severe disease, reduced responsiveness to standard therapies, and diminished quality of life. Although the underlying mechanisms remain incompletely understood, adipokines such as leptin and other inflammatory cytokines from adipose tissue may contribute to this link [20]. Whether these mediators play a pathogenic role or serve as useful biomarkers of disease activity and treatment response remains an open question. Our findings indicate that, at least inthe short term, high-dose ICSs do not significantly alter leptin levels or appetite in adults with stable asthma, including those who are overweight.

Importantly, these findings may hold particular relevance within the evolving framework of personalized medicine. While our study population exhibited a metabolically neutral response to short-term high-dose ICSs, it is increasingly recognized that interindividual variability—driven by both clinical and molecular factors—can influence corticosteroid sensitivity and susceptibility to adverse effects. For example, individuals with pre-existing metabolic syndrome, obesity, insulin resistance, or specific genetic polymorphisms (e.g., NR3C1 or FKBP5 variants) may exhibit altered glucocorticoid receptor activity, which could predispose them to more pronounced metabolic responses even with inhaled routes of administration [21]. The identification of such high-risk phenotypes could, in future, facilitate more nuanced prescribing strategies—adjusting ICS dose, delivery method (e.g., nebulized vs. extrafinepMDI), or duration based on personalized risk profiles.

Although no validated biomarkers currently exist to predict short-term metabolic responses to ICSs, future research may explore circulating adipokines (leptin, adiponectin), cortisol reactivity profiles, and multi-omic signatures (including transcriptomics and metabolomics) as candidate predictors. Our current findings in a well-characterized, metabolically stable cohort may serve as a reference phenotype for future comparative studies. Moreover, given that nebulized ICSs may differ in systemic absorption compared to extrafinepMDI formulations, targeted investigations into device-specific metabolic effects are warranted. Ultimately, integration of such biomarkers and individual risk factors into treatment algorithms could support a precision medicine approach—optimizing asthma control while minimizing side effect burden.

Several limitations of our study should be noted. The small sample size limits our ability to detect subtle metabolic effects. The 14-day duration may not reflect potential cumulative impacts of longer-term high-dose ICS use. Our participants had mild asthma, which may limit the generalizability of the findings to individuals with more severe disease or obesity, who may be more susceptible to metabolic disturbances. It is notable that the study population was, on average, overweight. As leptin levels are closely associated with adiposity, baseline leptin concentrations may have been elevated, potentially obscuring subtle treatment-related changes. Moreover, the generalizability of these findings to normal-weight individuals or those with obesity should be interpreted cautiously, and subgroup analyses by BMI category may be warranted in future studies. Lastly, while adherence was objectively verified, the observational design does not permit causal inferences.

Future studies should aim to address these limitations by enrolling larger and more diverse cohorts, including individuals with obesity and more severe asthma, and by extending the duration of follow-up to assess long-term metabolic outcomes. Comparative trials evaluating different ICS formulations and delivery devices could also be informative, as systemic bioavailability varies across products. Mechanistic studies exploring the effects of ICSs on other adipokines, appetite-regulating hormones, and central nervous system pathways would further enhance understanding in this area.

In conclusion, our pilot study provides reassuring evidence that short-term high-dose ICS therapy may not lead to significant changes in appetite, dietary intake, leptin levels, or body weight in adults with stable asthma. These findings complement existing data, contrast with the well-documented metabolic side effects of OCSs, and address a key patient concern. By offering objective data, our study may support improved adherence and better clinical outcomes in asthma management. Further research is needed to confirm these results across broader patient groups and over longer periods of ICS exposure.

## Figures and Tables

**Table 1:** Clinical characteristics of the study population, by gender.

	All (n = 35)	Male (n = 19)	Female (n = 16)
Age (years)	48.7 ± 15.1	48.7 ± 13.8	48.7 ± 17
FEV1 (% predicted)	89 ± 5.9	88.5 ± 5.9	89.6 ± 6.1
FVC (% predicted)	94.8 ± 6	93.3 ± 5.9	96.6 ± 5.8
FEV1/FVC (%)	0.75 ± 0.3	75.7 ± 3.1	75.2 ± 3.4
ACQ 6, med [IQR]	1.7 ± 0.5	1.6 ± 0.4	1.8 ± 0.5
Current smokers (%)	4 (11.4)	2 (10.5)	2 (12.5)
Ex-smokers (%)	5 (14.2)	2 (10.5)	3 (18.7)
Pack-years	3.57 ± 7.7	2.8 ± 7.5	4.5 ± 8.2
ICS * daily dose (μg) during the study	571.4 ± 66.7	579 ± 71.3	562.5 ± 61.9

Values represent means ± SD or number of subjects (%).Abbreviations used: FEV1, forced expiratory volume in 1 s; FVC, forced vital capacity; ACQ 6, 6-item Asthma Control Questionnaire; ICS, inhaled corticosteroid. * Extrafine beclomethasone dipropionate/formoterol.

**Table 2:** Effect of treatment on anthropometry, appetite, dietary intake, and leptin levels.

Outcome	First Visit (Baseline)	Second Visit (2 Weeks)	Mean Difference (95% CI)	*p* Value
Weight (kg)	85.90 ± 14	85.91 ± 14	+0.01 (−0.45 to +0.47)	0.901
Males (n = 19)	93.56 ± 11.1	93.62 ± 11.3	+0.06 (−0.12 to +0.24)	0.501
Females (n = 16)	76.8 ± 11.7	76.7 ± 11.4	−0.10 (−0.76 to +0.56)	0.807
BMI (kg/m^2^)	28.8 ± 3.7	28.8 ± 3.7	0.00 (−0.21 to +0.21)	0.912
Males (n = 19)	29.1 ± 3.1	29.1 ± 3.2	0.00 (−0.19 to +0.19)	0.554
Females (n = 16)	28.5 ± 4.4	28.5 ± 4.3	0.00 (−0.23 to +0.23)	0.869
Energy intake (kcal/day)	2391.5 ± 646.4	2426.1 ± 636.5	+34.6 (−40.9 to +110.1)	0.079
Males (n = 19)	2616.2 ± 653.6	2656.9 ± 621.9	+40.7 (−27.8 to +109.2)	0.152
Females (n = 16)	2124.7 ± 542.8	2152 ± 554	+27.3 (−30.0 to +84.6)	0.332
Energy intake (kcal/day/kg BW)	27.5 ± 3.1	26.9 ± 3.8	−0.6 (−0.89 to −0.31)	**0.042**
Males (n = 19)	27.6 ± 2.9	27.1 ± 3.9	−0.5 (−1.02 to +0.02)	0.065
Females (n = 16)	27.3 ± 3.5	26.6 ± 3.8	−0.7 (−1.61 to +0.21)	0.074
Appetite visual score	4 ± 0.6	4.1 ± 0.6	+0.1 (−0.11 to +0.31)	0.473
Males (n = 19)	3.9 ± 0.6	4 ± 0.6	+0.1 (−0.11 to +0.31)	0.205
Females (n = 16)	4.2 ± 0.6	4.2 ± 0.7	0.0 (−0.20 to +0.20)	0.806
Leptin (ng/mL)	10.4 ± 7.7	11.2 ± 9	+0.8 (−3.47 to +3.72)	0.207
Males (n = 19)	11.3 ± 9.1	11.2 ± 8.8	−0.1 (−3.50 to +3.30)	0.762
Females (n = 16)	9.3 ± 5.7	11.2 ± 9.4	+1.9 (−2.15 to +6.00)	0.174

Values represent means ± SD. Abbreviations used: BMI, body mass index; BW, body weight. *p* value was assessed using paired *t*-tests or Wilcoxon signed-rank tests as appropriate.

## Data Availability

The data supporting this study are not publicly available due to privacy and ethical restrictions.
